# Assessment of a change of protocol of prenatal screening by inclusion of non-invasive prenatal diagnosis

**DOI:** 10.1515/almed-2020-0011

**Published:** 2020-04-02

**Authors:** Rocío Cabra-Rodríguez, Guadalupe Bueno Rodríguez, Cristina Santos Rosa, Miguel Ángel Castaño López, Sonia Delgado Muñoz, Antonio León Justel

**Affiliations:** UGC de Análisis Clínicos, Hospital Juan Ramón Jiménez, Huelva, Spain; Servicio de Análisis Clínicos, Hospital Infanta Elena, Huelva, Spain; Hospital Juan Ramón Jiménez, Ronda Norte s/n, 21005, Huelva, Spain

**Keywords:** aneuploidies, non-invasive, prenatal diagnosis

## Abstract

**Objectives:**

Non-invasive prenatal screening (NIPS) is a test for the detection of major fetal chromosomal abnormalities in maternal blood during pregnancy. The purpose of this study was to assess the performance of NIPS implemented within the framework of the Screening Program for Congenital Abnormalities of the Andalusian Health System.

**Methods:**

A retrospective observational study was undertaken to determine the number of NIPS tests performed since its introduction. The number of invasive diagnostic tests done after the implementation of NIPS in the patients included in the program between March 2016 and August 2017 was also quantified.

**Results:**

A total of 6,258 combined first- and second trimester screening tests were performed, covering 95% of the population. In total, 250 subjects were identified as high risk, of whom 200 underwent NIPS after loss to follow-up. NIPS showed a sensitivity of 100% (95% CI: 76.84–100%) and a specificity of 99.46% (95% CI: 97.04–99.99%).

**Conclusions:**

This test has proven to have a very high sensitivity and specificity. The results obtained demonstrate that the incorporation of NIPS in clinical practice minimizes the rate of miscarriages and reduces the frequency of invasive procedures by 70%.

## Introduction

Fetal medicine currently faces prominent challenges including prenatal detection of genetic abnormalities causing major disabilities, among others. A variety of prenatal screening tests have been developed in the recent years. These new strategies include safe, easy-process, selective screening for fetal genetic abnormalities [1].

In the 70s, prenatal screening for fetal chromosomopathies was primarily based on maternal age. In the 80s, a set of biochemical markers were developed (alphafetoprotein and total or free β subunit of human chorionic gonadotropin [β-hCG]) and used in combination with maternal age to detect Down's syndrome (trisomies 21[T21]). However, this method was not very effective. New biochemical markers (pregnancy-associated plasma protein-A [PAPP-A]), and ultrasound markers (nuchal translucency [NT]) were incorporated to prenatal tests later. This way, detection rates increased to 85–90% for trisomy 21 [2].

In 2009, prenatal screening for chromosomal abnormalities was proposed to be incorporated in the Andalusian Health System (SSPA). This proposal adopted the recommendations of the Spanish Society of Gynecology and Obstetrics (SEGO). In addition, combined, first-trimester screening (first-T CS) was included in a larger scheme called the Andalusian Prenatal Screening Programme for Congenital Abnormalities (PACAC) [3], [4].

After the implementation of PACAC, a corporative application was incorporated to the SSPA (siPACAC) to facilitate risk assessment during the first (total and free PAPP-A, β-hCG and NT before 14 weeks gestation), and second trimester (free β-hCG and alpha-fetoprotein) for T21 and other trisomies [2], [3].

Data is processed by siPACAC, which yields a risk estimate. If risk is high, patients are offered an invasive procedure such as amniocentesis or chorionic villi sampling (CVS), with the risks that these procedures entail for the mother and the fetus [4], [5].

The rate of newborns with congenital defects is 2–3% at birth, of whom 1–1.5% are caused by malformations (60% of the total) and 0.5–1% (12–15%) by chromosomal abnormalities [6].

Confirmation of the presence of a chromosomal abnormality during the first and second trimester of gestation requires the analysis of genetic material. This material is obtained by invasive methods such as chorionic villi sampling or amniocentesis. However, these procedures are not completely safe and have an associated risk for fetal loss (approx. 1%). In addition, invasive methods have an added economic and emotional cost for the future mother. Informed consent is necessary for these invasive procedures, and it can be voluntarily revoked by the patient [4]. The most widespread screening tests for chromosomal abnormalities are conventional karyotyping and FISH or QF-PCR for chromosomes 13, 18, 21, X, and Y. Unlike conventional karyotyping, FISH or QF-PCR do not require previous fetal cell culture and provides results within 48 h [7].

New molecular biology techniques have been recently developed to add to screening for aneuploidies in pregnant women. This strategy involves non-invasive prenatal screening (NIPS). NIPS analyzes fetal DNA in maternal blood to assess the genetic characteristics of the fetus without entering the uterus; therefore, this method does not entail any risk for fetal loss [8].

This test detects major fetal chromosomic alterations in maternal blood, namely: chromosomes 21 (Down's syndrome), 18 (Edward's syndrome), 13 (Patau Syndrome) and X (Turner's syndrome). The sensitivity of NIPS for Down's syndrome is 99% and the rate of false positives is below 0.1% [9].

This test involves a simple analysis of maternal blood. It does not entail any risk either for the mother or the fetus and has a high diagnostic performance [10], [11].

The analysis of fetal DNA in maternal blood for the detection of trisomies 21, 18, and 13 is an effective but expensive diagnostic test that can be performed from the 10^th^ week of gestation. For this test to be more cost-effective, NIPS testing can be contingent on the results of the combined first-trimester test routinely performed in clinical practice [11], [12].

The proportion of fetal DNA (fetal fraction) in maternal plasma determines its reliability and should be taken into account in the interpretation of results. In general terms, the evidence published to date establishes 4% as the lower cut-off value to ensure a reliable result. If fetal fraction is <4% the sensitivity and specificity of the test decrease [13], [14]. However, positive NIPS needs confirmation by an invasive procedure, whereas negative results do not exclude chromosomal abnormalities at 100% [15], [16], [17], [18].

The objective of this study is to assess the efficacy of non-invasive diagnostic prenatal screening test (Harmony®). To such purpose, the sensitivity, specificity, and rate of false positives of the test were calculated in a population identified as high risk within the Andalusian Screening Program for Congenital Abnormalities.

## Materials and methods

A retrospective, observational study was conducted to determine the number of NIPS tests performed since its incorporation to the portfolio of services of a SSPA hospital in our healthcare district. The number of invasive diagnostic procedures (amniocentesis, chorionic villus sampling) after the incorporation of NIPS in patients engaged in the PACAC programme between March 2016 and August 2017 in Juan Ramón Jiménez hospital area was quantified. Recruitment was completed in August 2017. No further women were enrolled in the study, as it had to be ensured that gestation had come to full term. After birth, all newborns identified as high-risk for aneuploidies by combined first- and second trimester testing were examined.

This NIPS test (Harmony®) has been incorporated in our hospital as an initial test for fetuses with high-risk first- and second trimester test results requiring confirmation by an invasive procedure (amniocentesis, chorionic villus sampling).

**Figure 1:**
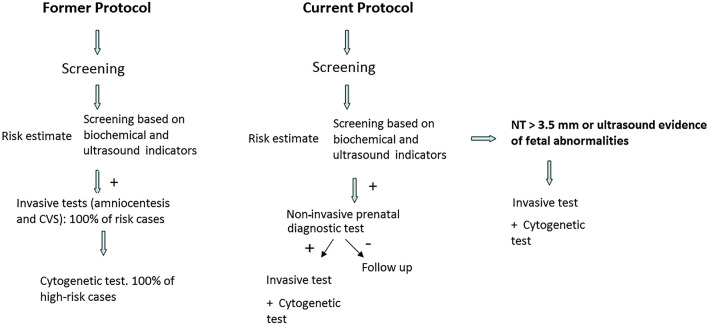
Diagnostic strategy with the incorporation of non-invasive prenatal diagnosis in our center.

In the presence of a high-risk first- and second trimester screening result for aneuploidies (≥1/280 for trisomy 21 and ≥1/150 for trisomies 13 and 18), the patient is offered NIPS as an intermediate screening test prior to an invasive procedure. When NIPS indicates a low risk (<1/10,000 = 0.01%), standard follow-up is adopted. If NIPS detects a high risk, the patient is offered an invasive procedure to confirm the alteration detected by means of a cytogenetic study (karyotyping and array CGH, where appropriate) (Figure 1).

In NIPS (Harmony®), maternal whole blood is drawn and sent to the external laboratory Megalab. To such purpose, informed consent is previously obtained from the mother. When the fetal fraction is not adequate (fetal fraction <4%), it is completed with a new blood extraction and determination. The results of this test are available within 7–9 work days.

This test is based on the analysis of circulating free DNA (cfDNA) in maternal plasma, of which 10% approximately is released from the placenta (non-exclusively fetal) [12]. Risk assessment for aneuploidies is based on the analysis of the total pool of maternal and placental DNA using several techniques (relative quantification by targeted microarray or genotyping) to obtain a final risk estimate [13], [19].

Patients identified as low-risk (<1/10,000 = 0.01%) by NIPS were followed-up for 25 months to estimate the sensitivity, specificity and rate of false positives of the test. The medical reports of all newborns were reviewed and none had any dysmorphic trait or phenotypic alteration either at birth or at discharge.

High-risk or >99% risk for aneuploidies according to NIPS were confirmed by karyotyping and/or microarray analysis of amniotic fluid. In turn, patients identified as low-risk by NIPS with normal ultrasound results underwent standard prenatal follow-up. All clinical records, obstetric reports and newborn examination results at birth and at discharge were reviewed. The number of patients who declined to undergo NIPS and an invasive procedure and the number of terminations of pregnancy due to ultrasound abnormalities without any supporting studies were recorded. Cases of spontaneous miscarriage or missed abortion and termination due to fetal malformations were considered a loss to follow-up and were not considered for statistical analysis. Patients offered NIPS who declined to undergo the test and newborns who were born in private centers and whose neonatal examination reports were not available were also considered a loss to follow-up in sensitivity and specificity calculations.

Demographic data and first- and second trimester aneuploidy screening test results were extracted from the siPACAC programme.

NIPS results (Harmony™) and other cytogenetic data were retrieved from the Infinity® Roche Diagnostics application and from the clinical records of patients.

Statistic analyses were performed using the MedCalc® Easy-to-use statistical software package, version 11.0.0.

## Results

**Figure 2:**
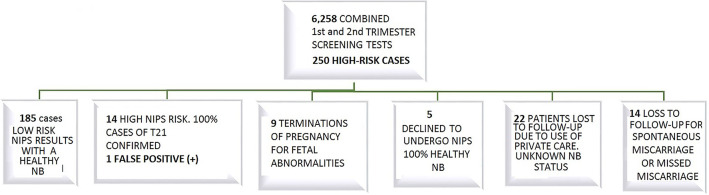
Results after the implementation of on-invasive prenatal diagnosis in our area.

A total of 6,258 combined first- and second trimester screening tests were performed, covering 95% of the population. A total of 250 patients were identified as high risk (≥1/280).During the first trimester, 211 screening tests yielded a high-risk result vs. 39 during the second trimester of gestation. Only a false-negative result was obtained from combined first-trimester aneuploidy screening, where NIPS was not subsequently performed. The sensitivity and specificity of NIPS was only estimated for patients with high risk by first- and second trimester screening for T21 associated with T13/T18 or both. The final number of cases analyzed was 200. Patients who underwent prenatal follow-up in private centers were not considered for analysis, as neonatal reports were not available. Cases of miscarriage, induced abortion and refusal to undergo NIPS were also not considered for analysis (Figure 2).

All high risk NIPS results (>99%) for T21 corresponded to singleton pregnancies. The totality of results was confirmed by karyotyping, except for a case, where the study was complemented with array CGH. No patients declined to undergo and invasive procedure after a high-risk NIPS result.

**Figure 3:**
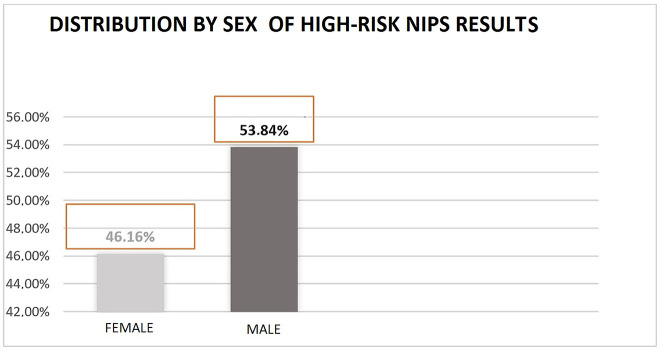
Sex-based distribution for non-invasive prenatal diagnosis result >99% for trisomy 21.

Sociodemographic and clinical data were characterized by: mean age of 36.06 years (18–44), smokers (30%), assisted reproductive fertility procedure (4%), diabetes mellitus (1.5%) and mean maternal weight in the first control prenatal visit of 67.3 kg (41–120). A complete summary of data is provided in Table 1 and Figure 3.

**Table 1:** Non-invasive prenatal diagnostic yield for trisomy 21 and trisomy 21 associated with Patau syndrome/Edwards syndrome.

Variable	First- and second-trimester
Number of patients	6,258 Cases of high risk based on NIPS results in 200
True positives	14
Negative positives	185
False positives	1
False positives	0
Sensitivity (95% confidence interval)	100% (76.84–100%)
Specificity (95% confidence interval)	99.46% (97.04–99.99%)
Positive predictive value (95% confidence interval)	93.33% (68.05–99.83%)
Negative predictive value (95% confidence interval)	100% (98.03–100%)
Prevalence of the disease	7% (3.88–11.47%)
Rate of false positives	0.53%

Of the nine induced abortions, two patients had undergone amniocentesis for PCR, karyotyping and array CGH for categorization of ultrasound alterations. The other patients did not undergo an invasive procedure due to serious malformations that made the fetus unviable and resulted in miscarriage. There was a confirmed case of Down's syndrome. In another case, diagnosis was not obtained despite the test was performed twice. In the presence of persistent hydrop-type ultrasound alterations, massive sequencing was performed and Noonan syndrome was confirmed.

A total of 18 amniocenteses were done during the study period: the two cases mentioned above and another 16 after indication based on a high-risk NIPS result. In contrast, 60 invasive procedures were performed during the same period the previous year prior to the incorporation of NIPS (March 2014, August 2015). Therefore, there was a 70% reduction in the frequency of invasive procedures. Finally, the rate of revocation to NIPS in our study was 2.85%.

## Discussion

The results obtained in our study show a very high sensitivity and specificity (99.46–100% respectively).

NIPS was found to have a high negative and positive predictive value (100–93.33% respectively). Thus, NIPS has proven to be excellent for the detection of chromosomal alterations, especially trisomy 21. This screening test is also effective for alterations in chromosomes 18 and 13 and has the potential to cover the entire genoma [20], [21].

The rate of false positives (1%) and the positive predictive value of NIPS (93.3%) (95% CI: 68.05–99.83%) slightly exceed those reported by Peral Camacho et al. [2], who reported a rate of false positives of 3.2% for a sample of 6,584 patients. Our results are also consistent with those obtained by other authors such as Norton et al. [9], who reported a rate of false positives of 0.06% and a positive predictive value of 80.9% (95% CI: 66.7–90.9%) for a sample of 15,841 patients.

The sensitivity and specificity of NIPS decreases in multiple pregnancies. However, only patients with singleton pregnancies were included in our study and no cases of multiple pregnancies were excluded [14].

This study has some limitations, especially a high rate of loss to follow-up due to continuation of prenatal follow-up in private centers, miscarriages, missed miscarriages, and abortions due to ultrasound abnormalities that could not be confirmed by the analysis of abortion specimens.

This study was not designed for comparison of combined first- and second-trimester screening and NIPS. Therefore, NIPS was not performed on the totality of the sample (n = 6,258), but only high-risk patients identified as such on combined first- and second trimester screening (n = 200), after exclusion of 50 cases of loss to follow-up. Further studies are needed involving a larger number of confirmed cases of chromosomal abnormalities that enable the generalization of results to other populations.

In conclusion, the incorporation of NIPS significantly reduces the frequency of invasive procedures and minimizes fetal loss and psychological stress, thereby improving routine clinical practice [11], [22]. A drawback to this technique is its high cost and limited availability, as not all laboratories perform this test [23]. In many countries, NIPS requires international shipping of samples, which may alter test results due to delays in preanalytical phase [8], [24].

The Department of Evaluation of Medical Products and Devices (SESC) of the Canarian Health System establishes the analysis of fetal DNA in maternal blood for the detection of trisomies 21, 18, and 13 as a second-tier or contingent prenatal screening test. Thus, SESC limits the use of NIPS to patients identified as high risk for fetal trisomy in chromosomes T21, T18 or T13 by combined first- and second trimester prenatal screening [25].

Standard criteria for the indication of NIPS should be established in our Public Health System. These criteria should include: cut-offs for definition of high risk, which is an indication of NIPS; the circumstances where NIPS is not recommended; limitations of the technique, and genetic counseling [26], [27].
